# Iatrogenic Femoral Artery Pseudoaneurysm (REVIEW OF TREATMENT OPTIONS)

**Published:** 2010

**Authors:** Omid Hashemi Fard

**Affiliations:** 1 Interventional Cardiologist, Isfahan Cardiovascular Research Center, Isfahan, Iran. Email: omidhashemifard@yahoo.com

**Keywords:** Atherosclerosis, Fibrinogen, Lovastatin, Rabbits

## Abstract

**BACKGROUND:**

Atherosclerosis, which is a result of gradual deposition of lipids in the lower part of blood vessel endothelium, is the leading cause of mortality and morbidity around the world. It has been proved that some inflammatory blood markers such as fibrinogen can predict the risk for cardiovascular disease conditions, not only in cardiovascular patients, but also in those who do not have any manifestations of the atherosclerotic development. In this study, the effect of cornus mas l. was evaluated on fibrinogen of hypercholesterolemic rabbits and it was also compared with lovastatin drug.

**METHODS:**

In this study, 25 New Zealand adult male rabbits were randomly divided into five groups of five. They were treated for 60 days by 5 different diets, namely basic, high cholesterol, regular plus 1 g/kgBW cornus mas L. powder, high cholesterol plus 1 g/kgBW cornus mas L. powder, and high cholesterol plus 10 mg/kgBW lovastatin. At the beginning and at the end of this period, blood samples were collected from the rabbits and their serum fibrinogen levels were measured.

**RESULTS:**

Cornus mas L. powder and lovastatin significantly decreased fibrinogen levels in comparison with high cholesterol group (P < 0.05). Furthermore cornus mas L. powder could reduce the fibrinogen level more than lovastatin (P < 0.05).

**CONCLUSION:**

The results indicated that consumption of cornus mas L. might be beneficial in atherosclerotic patients due to its reducing effects on fibrinogen.

## Introduction

Iatrogenic femoral artery pseudoaneurysm (FAP) forms when an arterial puncture site fails to seal (inability to apply hemostasis) allowing blood to jet into the surrounding tissues and form a pulsatile hematoma. These lesions lack a fibrous wall and are contained by a surrounding shell of hematoma and the overlying soft tissues. Pseudoaneurysms usually occur in low puncture situations, because there is suitable mass of soft tissue to contain the bleeding. In contrast, high puncture results in free hematoma due to lack of sufficient surrounding tissue. It can present as a new thrill or bruit, pulsatile hematoma, or marked pain or tenderness. Complications of pseudoaneurysms include rupture, distal embolization, local pain, neuropathy and local skin ischemia. Unlike FAPs of a surgical or post-traumatic nature, catheterization-induced pseudoaneurysm has usually a benign natural history and 80% of cases resolve spontaneously. Several therapeutic strategies have been developed to treat this condition. They include ultrasound-guided compression repair (UGCR), compression repair by mechanical devices, surgical repair, and minimally invasive treatments (thrombin injection, coil embolization and insertion of covered stents). UGCR of femoral artery pseudoaneurysm has become the first line treatment in many centers. The introduction of this procedure by Fellmeth in 1991 has reduced the need for surgical repair dramatically, and its cost is much lower than that of open and endovascular repair.^1^

## UGCR Technique

Ultrasound probe must be placed on the neck of pseudoaneurysm with the intention of complete obliteration of blood flow to the pseudoaneurysm. Sometimes, if the neck cannot be discerned precisely in ultrasonic evaluation, pressure is applied non-specifically on extraluminal lesion, but precise detection of pseudoaneurysm neck increases procedural success. Pressure is applied for a period of at least 1 minute, with the procedure repeated 10 times. At the end of each period, compression is relieved briefly to assess pseudoaneurysm patency and to reposition the transducer. Care must be practiced to avoid compromising flow within the underlying femoral artery. After successful thrombosis patients should lie supine for a few hours, with the affected leg in the stretched position.^2^–^4^ UGCR has considerable drawbacks including long procedure time, discomfort to patients and relatively high recurrence rate especially in patients receiving anticoagulant therapy (up to 35%). Moreover, UGCR has been shown to be less efficacious in patients with large FAP (i.e., larger than 3 to 4 cm in diameter), obese patients and those who cannot tolerate the associated discomfort. The procedure carries an overall complication rate of 3.6% and risk of rupture of 1%. Complications include acute pseudoaneurysmal enlargement, rupture, vasovagal reactions, deep vein thrombosis, atrial fibrillation and chest discomfort. Moreover, UGCR requires the availability of an ultrasound device and the presence of skilled personnel during the procedure.^2^–^4^

Compression on pseudoaneurysm of neck can be applied by mechanical devices like FemoStop.

## Compression Technique

Pseudoaneurysm of neck must be viewed precisely by ultrasonography and its location should be marked on groin skin. Also, neck compressibility must be tested by ultrasound probe. Then, device should be fixed on the exact location and pressure applied for 20 minutes intervals and each time, FPA neck patency evaluated by ultrasonography. If the neck was still patent, the procedure must be repeated. Some authors reached results comparable to UGCR in terms of feasibility, safety and effectiveness and better results in terms of operator and patient comfort and cost.^5^ Recently, para-aneurysmal saline injection has been advocated as a mean to treat FAP. It is a safe and effective technique comparable to UGCR. The procedure time is much lower in the latter and the patient is more comfortable.^6^

### Saline Injection Technique

Under sonographic guidance, 25–60 ml 0.9% normal saline must be injected in the tissues surrounding the tract connecting pseudoaneurysm to the femoral artery. Tract patency must be examined 24 hours later, sonographically.^6^

Thrombin injection is a more recent way to induce thrombosis in pseudoaneurysms. Thrombin is the active form of prothrombin. Fortunately thrombin that inadvertently leaks to the general circulation will rapidly diluted and antagonized by antithrombin and thrombomodulin.

## Thrombin Injection Technique

**Figure 1:**
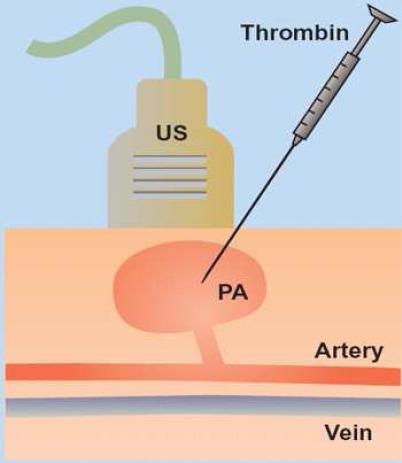
Ultrasound-guided puncture of pseudoaneurysm.

An ultrasound examination is used to define the relationship of pseudoaneurysm of neck and native vessels. The peripheral pulses are then documented. Under sterile conditions, a 22-gauge spinal needle is inserted with ultrasound guidance into the periphery of the pseudoaneurysm. The needle tip should be placed as remote from the pseudoaneurysm neck as possible. Human thrombin at strength of 1000 units/mL should be injected slowly via a 1 mL syringe. The pseudoaneurysm is scanned continuously and injection terminated when color flow ceases (Figure 1).^7^

In most cases, only 200 U (0.2 ml) thrombin is sufficient to induce thrombosis in the pseudoaneurysm. Rarely and especially in multiloculated cases, it becomes necessary to repeat injections. If most of the pseudoaneurysm became thrombosed but the flow in pseudoaneurysm of neck did not cease, it is not necessary to repeat injection. This will raise the risk of distal embolization and in most cases, flow in pseudoaneurysm of neck will cease in the later stages. At the end of procedure, peripheral pulses must be checked to exclude distal embolization. The patient must be in complete bed rest for 4 hours and scanned 24 hours later to confirm flow cessation in pseudoaneurysm. In another technique, thrombin can be injected directly in the pseudoaneurysm under fluoroscopic guidance while it is filled by contrast from the contralateral site. It is recommended to inflate a balloon catheter near pseudoaneurysm of neck to prevent thrombin leakage.^7^–^13^ More recently, collagen preparation has been used instead of thrombin with comparable results.

Complications of thrombin injection are rare and can be divided into allergic^10^, ^11^ and thrombotic reactions. While human thrombin usage has minimized the former, thrombotic accidents remains the well-known major complication. It occurs when large volume of thrombin is injected in a small pseudoaneurysm with a wide neck. In this case, thrombin may resolve spontaneously or may require intervention. Table 1 shows comparison of thrombin injection and UGCR in treatment of FAP.^7^, ^14^

**Table 1:** Comparison of thrombin injection and UGCR in treatment of FAP.

	Ultrasound-guided compression	Thrombin injection
**Procedure Time**	≥60 minutes	<15 minutes

**Pain**	Painful	Painless-local anaesthetic not required

**Intravenous sedation**	Frequently required	Not required

**Technical success**	74%^7^	93–100%^8^

**Effective with antiplatelet/anticoagulant agents**	Reduces efficacy	Yes

**Recurrence**	Up to 20%^9^	Rare

**Complications**	Rare	0–4%^9^

## Interventional techniques

Coil and Onyx embolization and stent graft implantation are two ways of treating arterial pseudoaneurysm. Due to efficacy of previously mentioned procedures, these procedures are seldom required.^15^, ^16^

## Coil Embolization Technique

Coil packing can be done from ipsilateral groin and directly in the pseudoaneurysm and rarely from the contralateral route by cross-over technique. In the former, coils are released one by one through a catheter with its tip placed directly in the pseudoaneurysm under fluoroscopic guidance. In another method, PDA coil was not released and remained attached to its delivery system, while thrombus formation was followed in the pseudoaneurysm. After pseudoaneurysm became completely thrombosed, coil, its delivery system and catheter were withdrawn from pseudoaneurysm.^15^

## Surgical treatment

**Figure 2:**
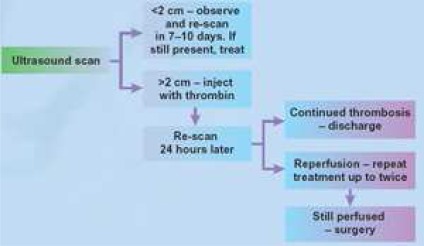
Suggested treatment algorithm of FAP.

It is beyond the scope of this article to discuss surgical techniques of pseudoaneurysm repair. But, it is worth to mention that surgery is now very seldom required in the management of post-catheterization FAP with its benign course and multiple nonsurgical therapeutic options. It must be reserved for large wide neck pseudoaneurysms which is often the result of trauma and surgery. Figure 2 depicts one treatment algorithm in the management of FAP.^7^
